# Effect of feeding sugarcane press mud on carcass traits and meat quality characteristics of lambs

**DOI:** 10.14202/vetworld.2015.793-797

**Published:** 2015-06-28

**Authors:** Ranjan Kumar, Subodh Kumar Saha, Sanjod Kumar Mendiratta

**Affiliations:** 1 Division of Animal Nutrition, Centre of Advance Faculty Training in Animal Nutrition, Indian Veterinary Research Institute, Izatnagar, Bareilly, Uttar Pradesh, India; 2 Division of Livestock Products Technology, Indian Veterinary Research Institute, Izatnagar, Bareilly, Uttar Pradesh, India

**Keywords:** carcass characteristics, lambs, sensory attributes, sugarcane press-mud

## Abstract

**Aim:**

To explore the possibilities of feeding unconventional agro-industrial byproduct for livestock production. Sugarcane press-mud (SPM), is a byproduct derived from sugarcane industry, which is rich in protein as well as minerals. The effects of dietary inclusion of SPM at different levels on the carcass characteristics of lambs were evaluated.

**Materials and Methods:**

SPM was incorporated in concentrate mixture at different levels 0% (SP_0_ - concentrate mixture without SPM [Control diet]), 10% (SP_10_ - concentrate mixture containing 10% SPM) and 20% (SP_20_ - concentrate mixture containing 20% SPM). The concentrate mixtures were fed along with wheat straw for 180 days. At the end of the experimental period, six lambs per group were slaughtered to evaluate carcass and meat quality characteristics.

**Results:**

No significant difference was observed in dressing percentage on pre-slaughter weight or empty body weight basis of lambs fed different levels of SPM incorporated diets. Likewise carcass weight, carcass length, and wholesale cuts appeared to have similar values among groups. The yield of visceral organs, chemical composition, and sensory attributes were not statistically affected by inclusion of SPM in the diets except juiciness of control group meat was significantly (p<0.05) higher than treatment group (SP_20_).

**Conclusion:**

The SPM can be incorporated in the diet of lambs up to the level of 20% without affecting the carcass characteristics of lambs.

## Introduction

Livestock is an integral part of the agriculture in India and could play an important role in future of the agricultural sector. This sector contributes approximately 25% of the value of output from total agriculture and allied sector. India hosts 65.06 million sheep accounting about 12.71% of livestock population that is a sizable livestock component requiring attention for effective feeding [1]. However, the continuous increase of human population and the liberalization of export policies have led to a shrinking of land size for growing of conventional feed and fodder used for livestock production. The requirement and availability of dry feed, concentrates, and green fodder most recently studied by Ministry of Agriculture, Government of India (GOI) [2]. According to the Ministry of Agriculture (GOI) [2], the deficit of dry fodder, green fodder and concentrates is 40%, 36%, and 57% respectively. The use of agro-industrial by-products could lead to a minimization of the gap between requirement and availability of feedstuffs for livestock feeding. Therefore, animal nutritionists are continuously in search of exploring the possibilities of feeding unconventional feedstuffs for livestock.

Sugarcane press-mud (SPM), a by-product of the sugarcane industry, is one such potential feed ingredient, which can be incorporated in livestock feeding. It is produced during clarification of sugarcane juice, and its current production is about 8-10 million tons [3] annually in India, which is roughly 23% of world’s production. Suresh and Reddy [4], Suma *et al*. [5] reported that SPM is a potential source of protein (11-13%) and other nutrients including major minerals Ca, P, K, Mg and S as well as trace minerals like Cu, Fe, Zn and Mn, which could contribute in body growth of animals. There are scarce data available regarding this aspect and there is not a scientific study referring to the dietary inclusion of SPM on lamb carcass characteristics.

Hence, the present study was conducted to evaluate the effect of graded level of sugarcane press mud in diet on carcass characteristics of lambs.

## Materials and Methods

### Ethical approval

This research work was carried out as per guidelines of the Institutional Ethics Committee, Indian Veterinary Research Institute, India. The experimental design and plan of the present study also duly approved by the academic council and Institutional Ethics Committee of Indian Veterinary Research Institute, India.

### Supply of SPM

The SPM was supplied by the Mirganj sugarcane factory, near Bareilly city. The freshly produced SPM was collected and dried under sun by repeated turning 3 times in a day, till it become air-dried and then was stored for experimental feeding and analysis.

### Formulation of experimental diets

Three iso-nitrogenous concentrate mixtures were formulated based on chemical composition of ingredients (Table-1). SPM was incorporated in concentrate mixture at different levels 0% (SP_0_, - concentrate mixture without SPM (Control diet), 10% (SP_10_ - concentrate mixture containing 10% SPM) and 20% (SP_20_ - concentrate mixture containing 20% SPM).

**Table-1:** Composition of experimental diets.

Particulars	SP_0_	SP_10_	SP_20_	SPM	WS
Physical composition (parts)					
Maize	44	43	41		
Wheat bran	27	19	13		
Soybean	26	25	23		
SPM		10	20		
Mineral mix.*	02	02	02		
Salt	01	01	01		
*Chemical composition (% DM)					
DM	95.34	95.70	96.12	89.87	94.97
OM	93.48	92.12	91.52	81.57	95.53
CP	20.55	20.73	20.64	16.22	3.42
EE	2.66	2.77	2.79	6.35	0.85
CF	4.12	5.10	5.62	13.65	46.16
NFE	66.15	63.52	62.47	45.35	45.10
Total ash	6.52	7.88	8.48	18.43	4.47
NDF	28.45	30.57	32.04	58.37	82.63
ADF	9.56	11.08	11.58	31.34	55.40
Hemicellulose	18.89	19.49	20.46	27.03	27.23
Cellulose	7.01	7.59	8.25	23.36	46.21
Calcium	1.04	1.32	1.72	5.07	0.51
Phosphorus	0.97	1.03	1.06	1.01	0.12

*Each values are mean of triplicate. DM=Dry matter, OM=Organic matter, CP=Crude protein, EE=Ether extract, CF=Crude fiber, NFE=Nitrogen Free Extract, NDF=Neutral detergent fiber, ADF=Acid detergent fiber, HC=Hemicellulose, SPM=Sugarcane press mud, WS=Wheat straw. *Chemical composition of mineral mixture (% by mass): Ca - 20, P - 12, Mg -5, Fe - 0.40, Cu - 0.10, I (as KI) - 0.026, Zn - 0.80, Co - 0.012, F - 0.07, Mn - 0.12

### Experimental animals and feeding

Twenty-one, 3-5 months aged Muzaffarnagri male lambs purchased from the local market with an average body weight of 11.70±0.29 kg were equally divided into three groups of seven animals in a completely randomized design. The lambs were housed in a well-ventilated clean barn having facilities for individual feeding and watering. All the lambs were reared under similar conditions throughout the experimental period of 180 days. The weighed amount of concentrate mixtures were offered daily in the morning at 9.00-9.30 AM to meet the protein requirement of a respective group of lambs as per ICAR requirement of sheep, goat and rabbit [6]. The rest of the nutrients requirements were met through *adlib* feeding of wheat straw. All the lambs were provided a fixed quantity of green fodder, twice a week to meet vitamin A requirement. Freshwater was offered *ad libitum*.

### Carcass traits

After 180 days of experimental feeding six animals from each group was slaughtered by *halal* method after overnight fasting with free access to water. Before slaughtering, the lambs were made unconscious by proper electrical stunning (0.6 A for 3 s) [7]. The dressing, evisceration, and legging were performed by adopting the standard procedure described by Gerrand [8]. After skinning and evisceration, the weight of hot carcass was measured. This was expressed as a percentage of live weight to estimate dressing percentage (DP). The carcass length (CL) was measured from the anterior edge of the aitch bone to the mid-point of the junction between the bodies of last cervical and first thoracic vertebrae [9]. Weight recorded after deducting gut fill from pre-slaughter weight (PSW) was noted as empty body weight (EBW). Carcass was divided into seven major primal cuts namely leg, loin, rack, breast and shank, shoulder, neck, and flank [10]. Weight of each cut was recorded and expressed as a percentage of carcass weight (CW). Loin eye area (cm^2^) was determined by tracing on a butter paper over the cut section of *Longissimus dorsi* (LD) muscle between 10^th^ and 11^th^ rib. The weights of edible and inedible viscera were recorded just after slaughter. From the standard leg cut of all the animals, meat and bone were separated and weighted to estimate meat-bone ratio. The pH of meat sample was determined after chilling (24 h) by blending 10 g of sample with 50 ml of distilled water for 1-min using a tissue homogenizer at 8000 rpm. The pH of the suspension was recorded by dipping combined glass electrode of a digital pH meter. Water-holding capacity (WHC) of meat was estimated by a centrifugation method [11]. The LD muscle of carcass was separately preserved by deep freezing (−18°C) for the analysis of chemical composition and sensory attributes of meat.

### Chemical analysis

The samples of SPM and feeds were analyzed for dry matter (DM), crude protein (CP), ether extract (EE), organic matter, crude fiber (CF), total ash (TA) and meat samples were subjected to chemical analysis for the estimation of moisture, EE, CP and TA on fresh basis as per standard procedure of AOAC [12]. Neutral detergent fiber (NDF) and acid detergent fiber were determined by the procedure of Van Soest *et al*. [13]. Nitrogen free extract (NFE) was obtained by deducting the sum of the percentage of CP, EE, CF, and TA on DM basis from 100. Calcium content in SPM and feed was analyzed by the procedure of Talapatra *et al*. [14]. The total inorganic phosphorus content in feed and SPM was estimated as per the standard procedure of AOAC [12].

### Sensory evaluation

Pooled LD muscle samples from each group were pressure cooked with salt (1.5% w/w) and without salt, and subjected to organoleptic evaluation on 8 point Hedonic scale (where eight is extremely desirable and one is extremely undesirable) by a panel of semi-skilled judges to evaluate appearance, flavor, taste, juiciness, texture, tenderness, and overall acceptability.

### Statistical analysis

**S**tatistical analysis of data was carried out according to procedures suggested by Snedecor and Cochran [15]. The collected data were subjected to statistical analysis using SPSS version 20 [16]. Means were compared using one-way analysis of variance test at 5% (p<0.05) level of significance using Duncan Multiple Range and F-test Duncan [17].

## Result and Discussion

### Chemical composition of SPM and concentrate mixture

The sun dried SPM employed in the present study contained 89.87, 16.22, 6.35, 13.65, 18.43 and 45.35 percent of DM, CP, EE, CF, TA and NFE respectively, besides 5.07 and 1.01 percent of calcium and phosphorus respectively (Table-1). From the proximate composition and minerals data, it appeared that SPM could substitute conventional feed ingredients like cereal crops in livestock nutrition. The above results were in agreement with finding of Ankita [18]. The higher EE content of SPM (6.35%), which might be due to presence of high wax content [4]. On the other hand, the calcium and phosphorus contents of SPM were slightly different from the composition reported by Suma *et al*. [19]. The variation in the chemical composition may be caused by the differences in quality of the cane crushed and the process used for clarification of cane juice in the sugar industry. The offered concentrate mixtures were iso-nitrogenous containing different levels of SPM; a progressive increase in the content of CF, NDF, TA and calcium with the increase of SPM levels in the concentrate mixture was observed. This was mainly due to the dietary inclusion of SPM since it contains a substantial quantity of CF, NDF, TA and calcium (Table-1). The above values and the ratio of Ca: P were within the normal range reported by Ankita [18].

### Slaughter and dressing characteristics

No significant differences were observed in PSW, EBW, CW, DP on PSW, EBW basis and CL of lambs fed different experimental diets (Table-2). This is in agreement with Suresh *et al*. [20] and Patel *et al*. [21], who also found no significant difference in DP of broilers and pigs, respectively fed sugarcane press-residue up to 10% level. The PSW did not affect DPs, as it is concluded by Vergara *et al*. [22]. Data of carcass traits (PSW, EBW, DP) indicated that there was numerically lower in SP_20_ compared to control and SP_10_ lambs (p>0.5). The CL and loin eye area of the carcass of different groups were ranged between 70.83 and 67.33 cm and 12.51 to 11.73 cm^2^ respectively, which were not significantly different among the groups. Loin eye area measured as an index of muscle growth was also no different among the experimental groups (p>0.05). Similar observations were also observed by Lade [23] in ram carcasses. The meat content and meat: bone ratios of standard leg cut were also similar among the experimental groups, although a marginal difference was observed (SP_0_ and SP_10_ vs. SP_20_ group). The meat: bone ratio of the carcass cuts increased as slaughter weight increased, however, that was statistically (p>0.05) similar across the diets. The ratio of meat: bone and edible: inedible portion of the carcass was not significantly different among experimental groups.

**Table-2:** Effect of feeding SPM diets on dressing characteristics and wholesale cuts of lambs.

Attributes	SP_0_	SP_10_	SP_20_	SEM*
PSW (kg)	26.23	26.05	24.09	0.51
CW (kg)	10.79	10.63	9.92	0.21
EBW (kg)	19.36	19.10	17.90	0.31
DP (% of PSW)	41.19	40.77	41.14	0.31
DP (% of EBW)	55.78	55.58	55.31	0.50
CL (cm)	70.83	70.66	67.33	0.89
Loin eye area (cm^2^)	12.51	11.98	11.73	0.15
Meat	2.88	2.85	2.66	0.06
Bone	0.99	1.03	0.99	0.01
Meat: Bone	2.90	2.76	2.67	0.04
Edible (% PSW)	53.84	53.15	53.26	0.41
Non-edible (% PSW)	27.08	27.91	28.27	0.36
Edible: Non-edible	1.99	1.91	1.88	0.03
Leg	35.96	36.66	36.91	0.20
Breast and Shank	20.77	20.44	19.99	0.22
Loin	7.74	7.43	7.48	0.12
Neck	8.47	8.38	8.49	0.13
Shoulder	8.30	7.89	7.02	0.27
Flank	2.62	2.62	2.76	0.18
Rack	15.09	16.01	15.62	0.22

PSW=Pre-slaughter weight, CW=Carcass weight, EBW=Empty body weight, DP=Dressing percentage, CL=Carcass length,

* SEM=Standard error mean, SPM=Sugarcane press mud

### Wholesale cuts

The weight of wholesale cuts, such as leg, breast and shank, loin, neck, shoulder, flank and rack expressed as a percentage of CW appeared to be similar among the groups of lambs (Table-2). This finding was in accordance with that of Sahu *et al*. [24], who also observed no significant differences in primal cuts from crossbred pigs fed with graded levels of SPM in diets.

### Visceral organs

The weight of liver, kidney, heart, testes, spleen, lungs with trachea, full gastrointestinal tract, skin, and blood, presented as percentage of PSW, did not significantly differ among groups (p>0.05) (Table-3). These results are in agreement with the observation of Budeppa *et al*. [25] who also reported that the weight of liver, heart and gizzard remained statistically similar (p>0.05) among the broiler birds fed with 4% level of SPM in diets. Similar results were also reported by Sahu *et al*. [24] from crossbred pigs carcass and further concluded that SPM did not influence any carcass traits including edible as well as inedible offals. The present finding was near to the observations of Suresh *et al*. [20], who reported that the weight of visceral organs such as liver, heart, gizzard, and spleen in broilers was not influenced by the dietary supplementation with sugarcane press residue.

**Table-3:** Effect of SPM dietary inclusion on weight of visceral organs of lambs, presented as percentage of preslaughter weight (% PSW).

Attributes	SP_0_	SP_10_	SP_20_	SEM
Edible				
Liver	1.34	1.33	1.33	0.02
Kidney	0.26	0.26	0.24	0.01
Heart	0.49	0.45	0.46	0.01
Testes	0.67	0.65	0.72	0.02
Head	7.19	7.03	6.77	0.14
Feet	2.70	2.63	2.59	0.04
Inedible				
Spleen	0.20	0.20	0.18	0.01
Lungs with trachea	1.23	1.31	1.31	0.03
GIT (full)	26.15	27.17	25.44	0.61
Skin	11.18	11.27	11.08	0.30
Blood	3.44	3.47	3.73	0.09

SPM=Sugarcane press mud, PSW=Preslaughter weight, GIT=Gastrointestinal tract, SEM=Standard error mean

### Chemical composition, quality characteristics and sensory attributes of meat

pH and WHC (%) of LD muscle ranged from 5.46 to 5.42 and 20.14 to 19.10 respectively, but did not significantly differ among the groups (Table-4). At the same time, muscle pH was not affected by slaughter weight. Santos *et al*. [26] also reported that slaughter weight neither affect pH nor its rate of postmortem decline as measured 60 min after slaughter and after refrigerated storage for 24 h. WHC is related with important meat organoleptic properties such as juiciness and tenderness [27]. The present results agree with the findings of Rajkumar *et al*. [28], who observed no significant differences among different slaughter weight groups of lambs in an intensive production system. In present study slightly higher values of WHC were observed in the control group (SP_0_). This may be attributed to the no significantly higher pH value. Meat with high WHC will hold more water resulting in low cooking loss [29].

**Table-4:** Effect of SPM dietary inclusion on quality characteristics, chemical composition and sensory attributes of *Longissimus dorsi* muscle of lambs.

Attributes	SP_0_	SP_10_	SP_20_	SEM
pH	5.46	5.44	5.42	0.02
WHC (%)	20.14	19.69	19.10	0.21
Chemical composition (% on fresh basis)				
Moisture	74.70	75.14	75.25	0.18
Protein	21.81	22.65	22.95	0.23
Fat	4.56	4.01	3.68	0.19
Ash	1.51	1.47	1.56	0.05
Sensory attributes				
Appearance	6.54	6.54	6.46	0.06
Flavor	6.50	6.36	6.25	0.07
Juiciness*	7.08^a^	6.93^ab^	6.63^b^	0.08
Tenderness	6.48	6.29	6.31	0.06
Overall acceptability	6.53	6.34	6.32	0.08

SPM=Sugarcane press mud, WHC=Water holding capacity, SEM=Standard error mean.

* Means in the row with the different superscripts are significantly different (p<0.05)

The chemical composition of LD muscle was similar among the experimental groups. However, EE of meat from the control group (SP_0_) was slightly higher (p>0.05) compared to that of treatment groups that may lead to lower CP. The loin eye area (12.51 cm^2^) and meat fat percentage (4.56%) was high in control than treatment groups but not at a significant level. Katole *et al*. [30] reached to the same conclusions since they did not find any significant difference in chemical composition of fresh meat of rams fed supplemented diets with different levels of Jatropha meal. Chemical compositions of loin eye muscle as also reported by Sahu *et al*. [24] were in agreement with present observations.

The sensory evaluation (appearance, flavor, tenderness and overall acceptability) of pressure cooked meat by a semi-trained panel revealed no significant differences between control and SPM groups (p>0.05) except from juiciness. Result of sensory attributes indicated that juiciness of control group was significantly (p<0.05) higher than treatment group (SP_20_) which might be due to higher content of fat and value of WHC in control meat.

## Conclusion

It is concluded that the inclusion of SPM up to 20% level in lamb’s diets has no any adverse effect on carcass and meat characteristics namely DP, wholesale cuts and chemical composition of meat.

## Author’s Contributions

SKS planned, guided and supervised the entire research work. RK carried out the experimental work and laboratory analysis. Manuscript preparation along with data analysis was done by RK. SKM has given dynamic suggestion during the carcass study. All authors read and approved the final manuscript.
